# Novel Modified Styrene-Based Microspheres for Enhancing the Performance of Drilling Fluids at High Temperatures

**DOI:** 10.3390/gels9090763

**Published:** 2023-09-19

**Authors:** Xianfa Zhang, Jingping Liu, Jinsheng Sun, Kaihe Lv, Zonglun Wang, Zhe Xu, Yuanwei Sun

**Affiliations:** 1 School of Petroleum Engineering, China University of Petroleum (East China), Qingdao 266580, China; mrzhangxianfa@163.com (X.Z.); lov3yo6@126.com (Y.S.); 2 CNPC Engineering Technology R & D Company Limited, Beijing 102206, China

**Keywords:** plugging, drilling fluid, polymer microsphere, deep oil and gas, high temperature

## Abstract

Ensuring wellbore stability is of utmost importance for safety when drilling in deep formations. However, high temperatures severely disrupt the drilling fluid gel system, leading to severe stability issues within ultra-deep formations containing micropores. This study focused on the development of a polymer-based plugging material capable of withstanding high temperatures up to 200 °C. A kind of microsphere, referred to as SST (styrene–sodium styrene sulfonate copolymer), was synthesized with a particle size of 322 nm. Compared to polystyrene, the thermal stability of SST is greatly improved, with a thermal decomposition temperature of 362 °C. Even after subjecting SST to hot rolling at 200 °C for 16 h, the particle size, elemental composition, and zeta potential remained stable within an aqueous dispersion system. The results of core displacement and NMR tests demonstrate that SST considerably reduces the pore diameter with a remarkable plugging efficiency of 78.9%. Additionally, when drilling fluids reach 200 °C, SST still enhances drilling fluid suspension and dispersion, and reduces fluid loss by over 36% by facilitating the dispersion of clay particles, improving the gel structure of the drilling fluid, resisting clay dehydration, and promoting plugging. The development of SST provides valuable insights into the preparation of high-temperature-resistant microspheres and the formulation of effective plugging agents for deep-well drilling fluids.

## 1. Introduction

Wellbore stability is a critical technical challenge that is commonly encountered in drilling engineering [1,2,3,4,5]. With the increasing exploration and development of oil and gas reserves in deep and unconventional formations, the issues surrounding wellbore stability in high-temperature and complex formations have become more severe [6,7,8,9], primarily due to the existence of substantial micro-pores within the surrounding rock formation near the borehole [10,11,12]. When the drilling fluid infiltrates the rock pores under pressure, the supporting force of the drilling fluid against the borehole wall decreases, causing clay minerals within the formation to swell through hydration. This, in turn, leads to problems such as borehole collapse, pipe sticking, and other drilling complications [13,14,15]. Borehole instability frequently occurs during drilling in shale and sandstone formations characterized by nano- and micron-sized pores. The filtration cake formed by the drilling fluid on the borehole wall is often unable to fully impede water flow into these pores [6,7,16]. Currently, plugging agents are widely employed in drilling operations to plug the rock pores, prevent the intrusion of the drilling fluid into the formation, and effectively guarantee drilling safety [17,18].

Currently, the existing plugging materials can be categorized into two types: rigid and flexible materials. Rigid plugging materials are typically inorganic, mainly ultrafine calcium carbonate, silicon dioxide, and graphene [19,20,21,22], which have demonstrated their ability to reduce the intrusion of drilling fluids into the formation by effectively plugging the pores and facilitating inter-particle aggregation. Moreover, they exhibit excellent high-temperature resistance. However, these rigid plugging materials often suffer from particle spacing issues [23], resulting in limited interaction between particles and between particles and the formation. Consequently, these materials tend to accumulate, become washed out, and possess an inadequate plugging effect [24]. Due to the limitations of rigid plugging materials, current research on plugging agents has shifted toward flexible plugging materials, including polymers and polymer-coated rigid plugging materials. The focus is on achieving self-adaptability of the plugging materials and improving the inter-particle and particle–stratum interactions [25,26].

In order to enhance the adaptability of inorganic plugging materials to the formation, many scholars have carried out the study of graft modification of inorganic materials. For example, microspheres of polymer-coated modified silica and microspheres of silane-graft-modified silica have dramatically improved the plugging efficiency of inorganic materials and have other additional effects such as lowering the filtration loss of drilling fluids and inhibiting shale swelling [27,28]. It is worth noting that for polymer-coated inorganic materials used as plugging agents, the addition of polymers often leads to a significant decrease in the high-temperature resistance of the inorganic microspheres, even below 150 °C. Additionally, the preparation process of these plugging agents tends to be complex, and their particle plugging performance heavily relies on rigid materials, lacking self-adaptive capabilities.

In recent years, there has been a growing interest in polymeric plugging agents as potential materials for drilling fluid plugging [29,30]. Polymer plugging materials possess deformability and adaptivity, allowing them to effectively block pores in the formations and contribute to the rheology, filtration loss, and high-temperature stability of drilling fluids [31,32]. Among these materials, styrene-based microspheres have emerged as promising plugging agents due to their easy preparation and tunable performance capabilities. For example, the microspheres of sulfonated polymer-coated styrene, tetramer microspheres, double-crosslinked styrene microspheres, and so on, have achieved efficient plugging in drilling fluids at 180 °C [33,34,35]. However, polystyrene is prone to decomposition at high temperatures, resulting in drastic changes in particle size for this type of microsphere at high temperatures as well, thereby reducing the dispersion stability and the overall performance of the drilling fluid [31,35]. As the drilling depth increases, the formation temperature increases to 200 °C or even higher. Consequently, the lack of high-performance plugging materials capable of withstanding such high temperatures has significantly restricted the development of ultra-deep drilling.

In this study, a new method for the preparation of microspheres by the copolymerization of styrene with sulfonated styrene was proposed to address the issue of high-temperature instability at 200 °C and provide new perspectives on the preparation of high-temperature resistance microspheres. The proposed plugging agent (SST) not only can plug formation micropores and reduce fluid loss but also significantly promotes the high-temperature stability of the drilling fluid gel system. The development of this plugging agent is expected to contribute to resolving the challenge of high-temperature resistance in drilling fluids and provide insights for preparing new microspheres with high-temperature stability.

## 2. Results and Discussion

### 2.1. FTIR

**Figure 1:**
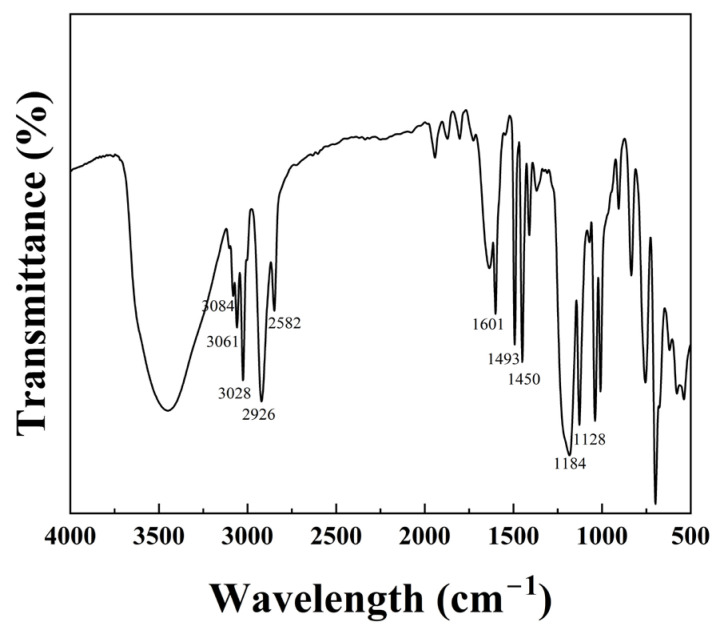
The infrared spectrum of SST.

In Figure 1, the peaks observed at 3084 cm^−1^, 3061 cm^−1^, and 3028 cm^−1^ correspond to the benzene ring–CH stretching vibration absorption peaks [24]. Additionally, the peaks at 2926 cm^−1^ and 2582 cm^−1^ represent the methylene-asymmetric and para-stretching vibration absorption peaks, indicating the polymerization of C=C double bonds. The peaks at 1601 cm^−1^, 1493 cm^−1^, and 1450 cm^−1^ are C–C stretching vibration absorption peaks in the benzene ring. Furthermore, the characteristic peaks of sodium styrene sulfonate are located at 1184 cm^−1^ and 1128 cm^−1^ [2], which represent the asymmetric and symmetric stretching vibration peaks of the sulfonic acid group, respectively. The IR spectrum confirms the successful polymerization of styrene-core microspheres with sodium p-styrene sulfonate and the SST is a copolymer of the two materials.

### 2.2. Thermal Gravimetric Analysis

**Figure 2:**
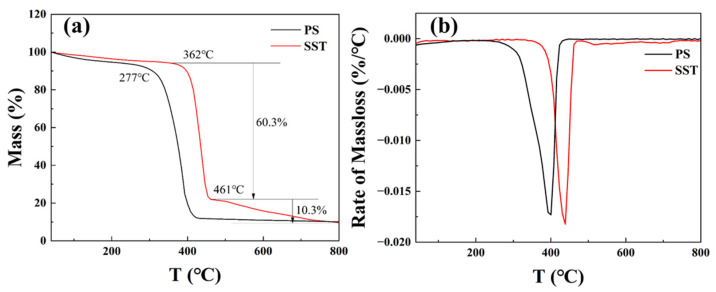
Analysis of the thermal stability ((**a**) Massloss; (**b**) Rate of massloss).

In order to compare the thermal stability of SST, polystyrene microspheres (PS) without sodium p-styrene sulfonate were prepared using the same method, and the thermal stability of the two materials is shown in Figure 2.

The thermal properties of SST were investigated through thermal gravimetric analysis. Figure 2a indicates that the thermal stability of SST is significantly higher than that of PS, and the initial thermal decomposition temperature is increased from 277 °C to 362 °C. As shown in Figure 2b, the rates of maximum thermal decomposition for both microspheres are comparable, characterized by a single main peak indicating intense decomposition upon reaching a critical temperature. However, the thermal decomposition of SST comprises two stages, which are the fast decomposition stage (362–461 °C) and the slow decomposition stage as temperatures larger than 461 °C due to the thermal degradation of the benzenesulfonic acid backbone. The observation further confirms the improvement in the thermal stability of the SST skeleton.

Since SST is stored in aqueous dispersion systems and primarily used in such environments, it is important to study its thermal stability in an aqueous dispersion system at a concentration of 2% (*v*/*v*).

### 2.3. Stability Analysis of Aqueous Dispersion Systems

The properties of SST, both before and after high-temperature treatment, were investigated by dispersing SST in water and subjecting the dispersion system to hot rolling at 200 °C for 16 h.

#### 2.3.1. Particle Size

**Figure 3:**
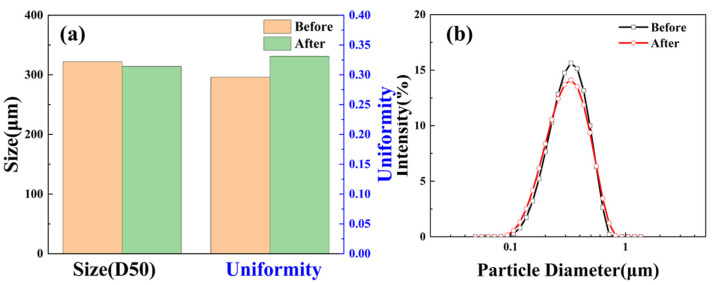
Particle size distribution of SST ((**a**) D50 and uniformity; (**b**) Particle diameter).

The particle size distribution of SST in the aqueous dispersion system, before and after hot rolling at 200 °C, is shown in Figure 3.

The median particle size (D50) of SST changes minimally, from 322 nm to 314 nm, following the high-temperature treatment. This indicates that its particle size remains highly stable at 200 °C. However, there is a reduction in particle size uniformity, leading to a wider particle size distribution as depicted in Figure 3b. This broader distribution is advantageous for sealing formations. At high temperatures, the SST particle size ranges from less than 100 nm to 1 micron, providing a wider operating range and facilitating particle size grading to improve plugging effectiveness.

#### 2.3.2. Micromorphology

**Figure 4:**
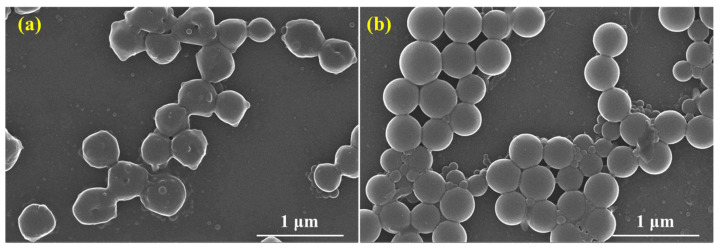
Microscopic morphology of SST ((**a**) Before; (**b**) After).

The shape evolution of SST before and after hot rolling was observed by SEM, and the microscopic morphology of SST is illustrated in Figure 4.

The morphology of the original SST is shown in Figure 4a. The microspheres with a rough surface are aggregated together, with smaller balls adhering to larger ones. After hot rolling at 200 °C, the surface of the microspheres becomes smooth and regular-spherical, and the adhesion force between the microspheres is significantly decreased due to the change in polarity at high temperatures. Consequently, the microspheres transform into regular spheres and dislodge the small microspheres from the surface of other microspheres, indicating that high temperature decreases the adhesion of the microspheres surface. In addition, an increase in microspheres dispersion results in a broader particle size distribution. Hence, high temperature facilitates the dispersion of SST, which is beneficial for its application under high-temperature conditions.

#### 2.3.3. Composition and Dispersion Stability

To study the dispersion properties and structural stability of SST, the zeta potential of SST dispersions was measured after hot rolling at different temperatures to evaluate the stability of SST in high-temperature aqueous dispersion systems. Moreover, the impact of high temperature on the composition of SST was analyzed by freeze-drying the dispersions and determining their elemental contents.

**Figure 5:**
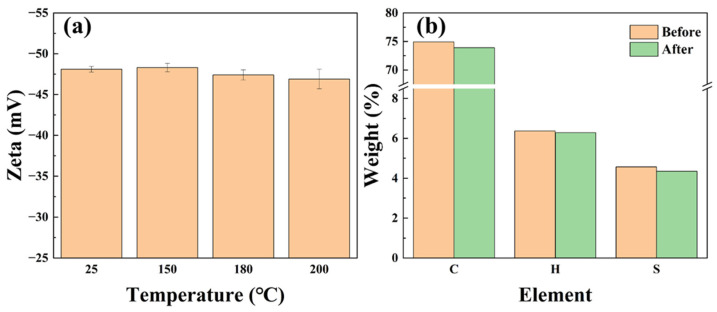
Zeta potential and composition of the SST dispersion system ((**a**) Zeta; (**b**) Element).

SST demonstrates excellent thermal stability in aqueous dispersion systems. Figure 5a displays the zeta potential of SST under different conditions, with the absolute value of the zeta potential serving as an indicator of dispersion system stability. The zeta potential of SST remains nearly constant following hot rolling for 16 h at various temperatures. The absolute zeta potential decreases from 48.1 mV to 46.9 mV after hot rolling at 200 °C but still at a high value, indicating that the stability of dispersion is excellent at 200 °C. The elemental proportions of SST remain at the same level after hot rolling at 200 °C, as shown in Figure 5b, confirming the high-temperature stability of SST from the composition structure. The above results indicate that SST can maintain the stability of its structure and surface properties and function effectively at 200 °C. Moreover, the proportion of element S in the feed was 4.8%, which was consistent with the ratio in the product, confirming the completion of the synthesis reaction and the formation of the desired SST product.

### 2.4. Application in Drilling Fluids

#### 2.4.1. Plugging Performance

Plugging micropores is a severe challenge in drilling engineering as it is essential to maintaining the stability of the borehole wall and drilling safety. Excellent plugging performance prevents the drilling fluid from invading the formation, avoids the shale’s hydration, and contributes to the wellbore stability. Therefore, the performance of SST in plugging was evaluated using NMR technology.

**Figure 6:**
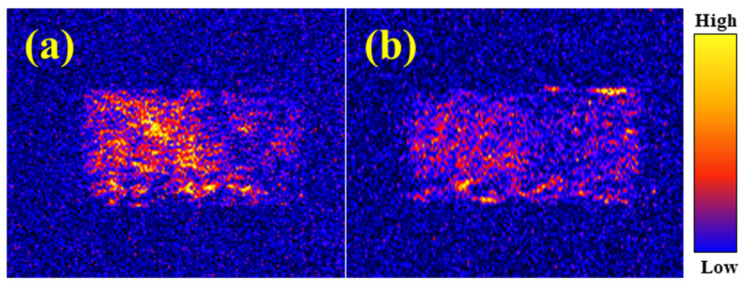
NMR images of cores ((**a**) Before; (**b**) After).

**Figure 7:**
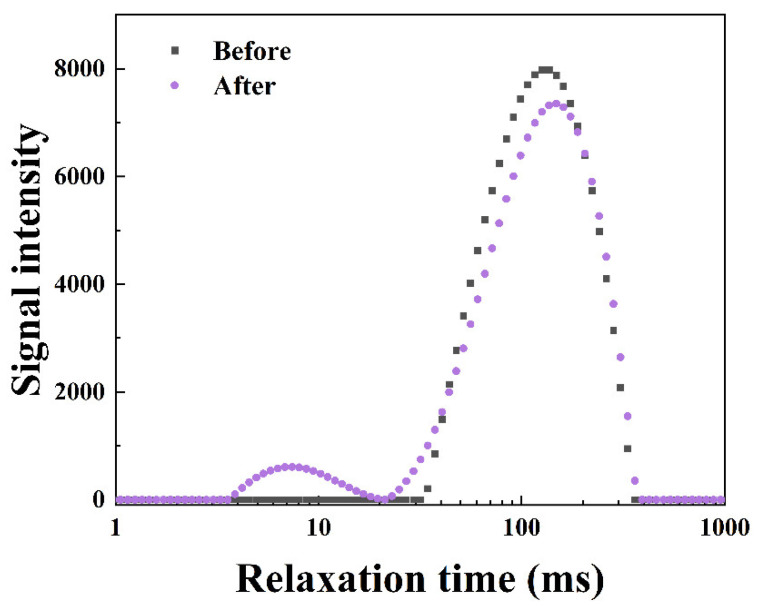
NMR T2 response curves of cores.

The morphology and NMR T2 curves of the artificial core before and after SST flooding are shown in Figure 6 and Figure 7.

Figure 6a illustrates the NMR pattern after the core was fully saturated with water, where the darker yellow color represents higher water content in the core. Because water only exists in the pores of the core, the color of the core can indicate the pores in the core. As shown in Figure 6b, the color of the core is more blueish compared to the core after water flooding, indicating that the number of pores saturated with water significantly decreases after sealing by SST. In addition, the permeability of the core reduces from 123 mD to 26 mD, which confirms that SST possesses an excellent plugging effect with a plugging efficiency of 78.9%.

The T2 relaxation time is positively correlated with the volume of the pores. Thus, the small T2 relaxation time corresponds to the small volume of pores in the core and vice versa [36]. The vertical axis indicates the water volume of the corresponding pores. Figure 7 illustrates the NMR T2 response curves for the core, and an apparent water volume reduction is observed in the core after plugging. Furthermore, the new peak appears near 8 ms in the curve, indicating smaller pores existing in the core. SST can effectively block the larger pores of the core, causing a new peak showing at a relaxation time nearly 10 times lower than the original peak and a significant reduction in the pore diameter of the core. Because the plugging agent SST accumulates near the inlet of the core, it fails to penetrate deeply into the core to plug all the pores, resulting in more water-bearing pores remaining in the pore space. Although a higher value of the peak exists in Figure 7, SST achieved an excellent sealing efficiency of 78.9%.

#### 2.4.2. Rheological Properties

**Figure 8:**
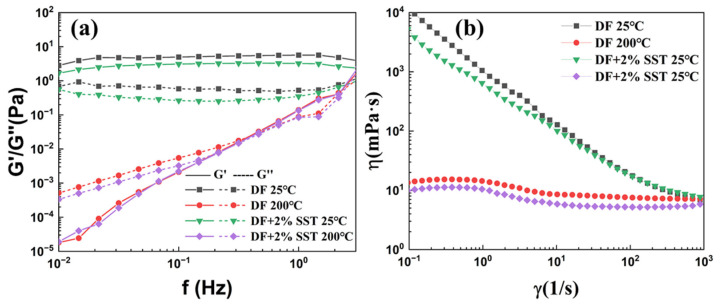
Rheological properties of drilling fluids ((**a**) G′ and G″; (**b**) viscosity).

Figure 8a presents the relation between the modulus and shear frequency of the drilling fluid. At room temperature, the drilling fluid gel system is stable, and inter-particle interactions are intense, resulting in constant G′ and G″ at this shear frequency. Because the gel structure is formed by the edge-to-edge and edge-to-face linkages between the bentonite in the drilling fluid, the drilling fluid exhibits more elasticity than plasticity: G′ > G″. However, after hot rolling at high temperatures, the hydration capacity of the clay surface decreases, inter-particle repulsion weakens, and the inter-clay mesh structure is severely damaged, leading to completely different rheological properties of the drilling fluid from those at room temperature.

The addition of SST slightly decreases the G′ and G″ of the drilling fluid before and after the hot rolling. The negative charge on the surface of SST and the electrostatic repulsion reduce the electrostatic attraction of the inter-clay structure and strengthen the mesh gel structure. However, adding SST has a minimal effect on the rheological properties of the drilling fluid, which can be seen in Figure 8b. SST has a negligible impact on the viscosity of the drilling fluid and does not alter its original rheological properties. In contrast, SST enhances the stability of the drilling fluid, which is further demonstrated in the subsequent sections.

#### 2.4.3. Fluid Loss Reduction

**Figure 9:**
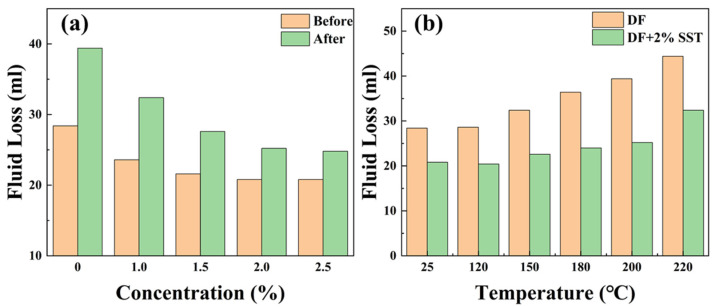
Fluid loss of drilling fluids ((**a**) Different concentrations of SST; (**b**) Different hot rolling temperatures).

The filtration performance of drilling fluids is critical for maintaining borehole wall safety. Generally, the plugging effect of the plugging agent can be evaluated by the reduction in drilling fluid filtration loss in combination with clay. SST was added to the drilling fluid, and the fluid loss of each drilling fluid before and after hot rolling at 200 °C was evaluated; the results are shown in Figure 9.

Figure 9a shows that the addition of SST can significantly reduce fluid loss. The fluid loss of the drilling fluid decreases gradually with the concentration of SST, and at the same time the reduction in the fluid loss becomes smaller. The optimum concentration of SST is 2%, considering both economy and effectiveness, which reduces the fluid loss before and after hot rolling at 200 °C from 28.4 mL and 39.4 mL to 20.8 mL and 25.2 mL, respectively, with a reduced rate of 26.8% and 36.0%. However, the fluid loss of the SST drilling fluid significantly increases at 220 °C, which indicates that severe damage occurs in SST when the temperature is beyond 220 °C. Although the damaging effect of high temperatures on the clay has an increased drilling fluid filter volume, SST can effectively reduce fluid loss under extreme conditions. Thus, SST has an excellent synergistic effect with the clay, which is attributed to the negatively charged SST promoting clay dispersion. At the same time, SST and clay form an excellent particle size grading and stack with each other to form a denser filter cake and decrease the filtration loss. In addition, SST has a better filtration loss reduction rate in the drilling fluid after high-temperature hot rolling, such as at a concentration of 2% SST. The particle size range of SST broadens at high temperatures and can better match the diversity of filter cake pores. Consequently, SST can better plug the pores of the filter cake and reduce fluid loss. The filter cake of the drilling fluid was observed using a scanning electron microscope, and the synergistic effect of SST and clay was also analyzed.

#### 2.4.4. Microscopic Morphology of the Filter Cake

**Figure 10:**
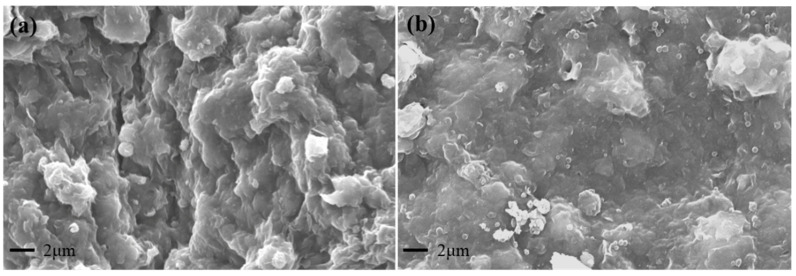
Microscopic morphology of drilling fluid filter cake ((**a**) Without SST; (**b**) With SST).

After drying the filter cake of the drilling fluid following hot rolling at 200 °C, the microstructure was investigated using SEM, and the images are shown in Figure 10.

The drilling fluid filter cake after hot rolling shows apparent agglomerated clay and crevices, and the surface of the filter cake is rough, as shown in Figure 10a [37,38]. When there are pores between clay particles, water easily flows out from these pores under the pressure difference of the drilling fluid, leading to an increased fluid loss and jeopardizing formation safety. After adding SST to the drilling fluid, the filter cake becomes flatter, and the size of large particles is significantly decreased, resulting in the reduction in fluid loss [37]. SST is interspersed between the clay particles to seal the pores of the filter cake and prevent the drilling fluid from invading the formation. Meanwhile, SST is included in the clay that is dispersed in the drilling fluid with small particle sizes, which is beneficial for clay stacking to form a dense filter cake. These observations prove the decent synergistic effect between SST and clay. In addition, the mechanism of interaction between SST and clay is revealed by the stability analysis and particle size distribution of the drilling fluid.

#### 2.4.5. Stability Analysis

The stability of drilling fluids, including suspension stability and dispersion stability, is the basis for the comprehensive performance of drilling fluids. Therefore, the stability of the drilling fluid was evaluated through experiments regarding colloidal stability and suspension stability, and the contribution of SST to the stability of the drilling fluid was analyzed through the experimental results.

**Figure 11:**
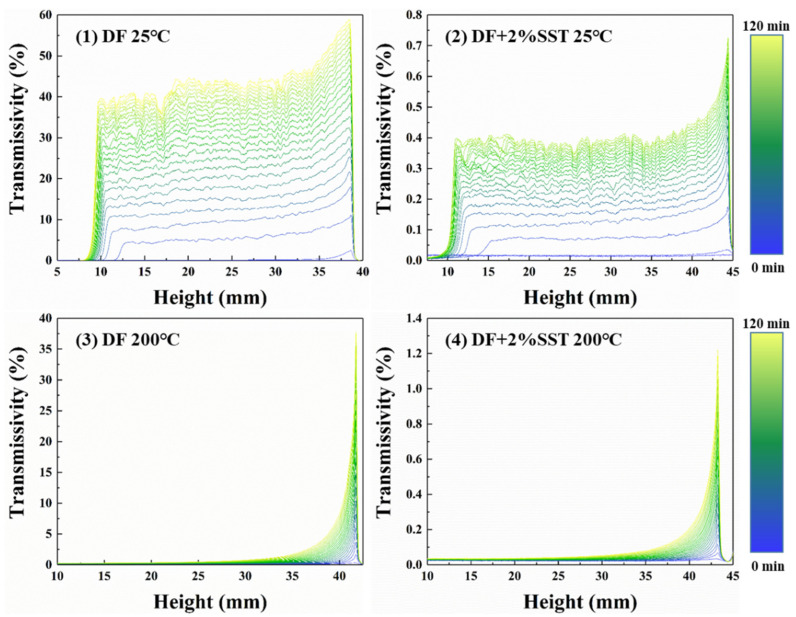
Suspension stability of the drilling fluid ((**1**) DF; (**2**) DF with SST; (**3**) DF after hot rolling; (**4**) DF with SST after hot rolling).

The drilling fluid is an opaque dispersion gel system; the higher the transmissivity, the greater the system deposition. The left, middle, and right sides of the curves in Figure 11 correspond to the drilling fluid’s bottom, middle, and top. As shown in Figure 11, the suspension stability of the drilling fluid at room temperature is poor. The highest transmissivity occurs at the top of the drilling fluid, indicating the highest sedimentation rate. The middle part of the drilling fluid exhibits a more uniform and slightly slower sedimentation rate compared to that of the top part. Despite the high temperature compressing the thickness of the clay hydration film and reducing intergranular forces, its effect of promoting clay dispersion is still greatly enhanced, as shown in Figure 11(3). After the drilling fluid experiences hot rolling, the high temperature enhances molecular thermal movements, hydration of the clay, and suspension stability, resulting in a sedimentation tendency at the top of the drilling fluid.

The addition of SST entirely changes the shape of the curve and improves its stability dozens of times due to the negatively charged nature of both SST and clay particles. Adding SST increases the electrostatic repulsion between the clay particles, thus improving the dispersion and suspension stability. The zeta potential measurements further confirm these stability improvements.

**Figure 12:**
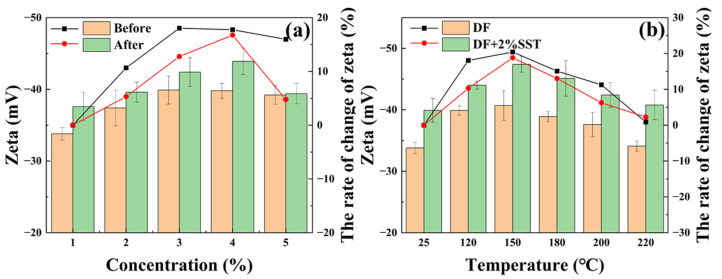
Zeta potential of drilling fluids ((**a**) Different concentrations of SST; (**b**) Different hot rolling temperatures).

As shown in Figure 12a, the zeta potential of the drilling fluid is about −35.2 mV at room temperature. SST can increase the absolute zeta potential value of the drilling fluid, and the absolute zeta potential increment increases as concentration increases. Thus, SST facilitates the clay’s dispersion and enhances the drilling fluid’s stability [39]. After hot rolling at 200 °C, the zeta potential trend of the drilling fluid impacted by SST is consistent with the trend at room temperature, and the zeta potential values are more significant than those at room temperature (maximum −43.9mV). As the concentration of SST reaches 2.5%, the stability of the drilling fluid decreases, possibly due to flocculation caused by excessive negative charges.

The impact of 2% SST on the zeta potential of the drilling fluid at different temperatures is shown in Figure 12b. A high temperature could promote the molecular movement of the clay, which is suitable for the hydration and dispersion of clay and the stability of the drilling fluid. However, a further temperature increase damages the clay’s hydration film, causing clay aggregation and reducing the drilling fluid stability. Meanwhile, SST can increase the zeta potential values of the drilling fluid, maintaining its hydration film thickness and promoting the clay dispersion to resist the damaging effects of high temperatures on the drilling fluid.

#### 2.4.6. Particle Size

**Figure 13:**
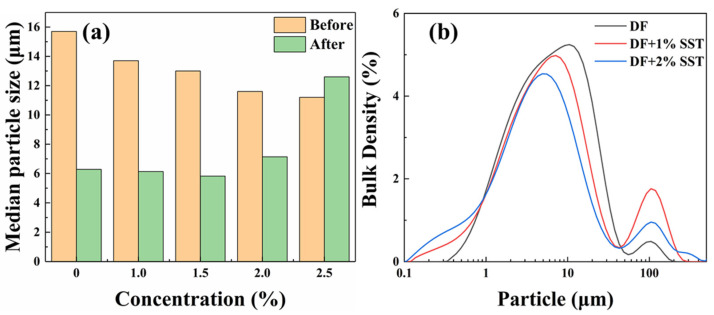
Particle size distribution of drilling fluids ((**a**) Different concentrations of SST; (**b**) Size distribution).

The mechanism of SST in the drilling fluid was analyzed through particle size testing, and the results are presented in Figure 13.

The median particle size of the clay, as shown in Figure 13a, provides the particle size variation in the drilling fluid. The particle size of the drilling fluid decreases with the increase in SST concentration at room temperature, demonstrating that SST facilitates clay dispersion. In addition, the hot rolling causes sufficient clay dispersion and significantly decreases the particle size of the clay. Although the high temperature promotes the dispersion of the clay, it dramatically compresses its diffuse double layer to reduce the inter-particle force (see Figure 12b). Consequently, the high temperature leads to a loose structure of the filter cake that cannot hold high pressure, increasing fluid loss. In contrast, SST improves the dispersion of the clay, but more crucial is that it protects and enhances clay hydration and reduces the damaging effect of high temperature on clay, thus improving the dispersion stability of the drilling fluid. The multi-faceted beneficial effects of SST confirm that SST can comprehensively enhance drilling fluid performance at high temperatures.

The application of the plugging agent SST in drilling fluids at 200 °C significantly improved the stability of the ultra-high temperature drilling fluids. The styrene-based microspheres can be highly stable by copolymerizing with sodium styrene sulfonate. It shows the feasibility of the microspheres copolymerized with styrene and sodium styrene sulfonate as ultra-high-temperature agents for drilling fluids.

## 3. Conclusions

In this study, high-temperature-resistant styrene-based microspheres were prepared, and their characteristics and effects on drilling fluids were investigated. The key findings are listed as follows.

1. A kind of monodisperse hydrophilic microsphere with a particle size of 322 nm was prepared by the copolymerization of sodium styrene sulfonate and styrene, which exhibited a significant core-plugging efficiency of 78.9% and effectively reduced the rock pores.
2. Unlike general polystyrene-based microspheres, SST has excellent thermal stability, and it can maintain its stability of particle size, morphology, elemental composition, and zeta potential in an aqueous solution at 200 °C.
3. For drilling fluids under 200 °C, SST still improved the filter cake quality, enhanced suspension and dispersion stability, and reduced fluid loss by more than 36%.
4. It was found that in drilling fluids, SST achieved improvement in drilling fluid performance in multi-functional ways such as by plugging the micropores of the filter cake, improving the dispersion of the clay, and promoting the particle size grading of the drilling fluids.

## 4. Materials and Methods

### 4.1. Materials

Styrene (St, 99%) and ammonium persulfate ((NH_4_)_2_S_2_O_8_, AR) were purchased from Sinopharm Reagent Co. Sodium p-styrene sulfonate (SSS, 90%) and sodium carbonate (99.8%) were provided by Shanghai Maclean Biochemical Technology Co., Shanghai, China. Polyethylene glycol octylphenyl ether (OP-10, 99%) was obtained from Beijing J&K Science Co., Beijing, China. Bentonite is an industrial product from Huaian County Tengfei Bentonite Development Company, Ltd., Zhangjiakou, China. The based bentonite fluid (DF) was made by adding 4 wt% bentonite and 0.3 wt% sodium carbonate to water and stirring for 24 h [2,8,9].

### 4.2. Preparation of SST

**Figure 14:**
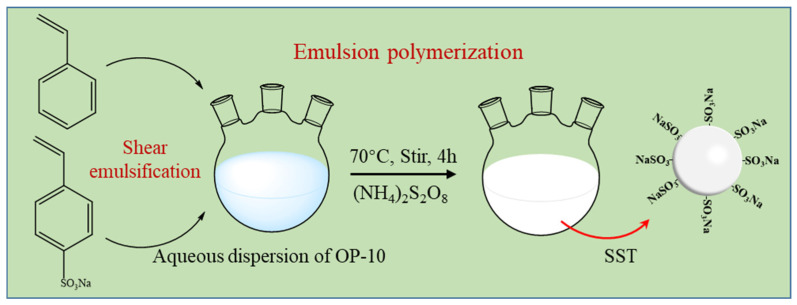
Synthesis pathway of SST.

The synthesis pathway of SST is illustrated in Figure 14, and the procedure for preparing SST is as follows:

Adding 100 mL of deionized water to a beaker and dispersing OP-10 into the water, then adding 45 g of St and SSS with a mass ratio of 2:1 to the beaker.

Shearing dispersion emulsifying at 2000 r/min for 10 min, followed by transferring the mixture to a three-necked flask and heating up to 70 °C in a water bath.

Stirring the dispersion at 800 r/min using magnetic stirring and de-oxygenating it with nitrogen for 30 min.

Dissolving 0.225 g ammonium persulfate in 2 mL of water and gradually adding it to the dispersion at a constant temperature for 4 h.

Obtaining a white dispersion system of microspheres SST.

The SST used for physicochemical property analysis was dialyzed through dialysis bags under a deionized water environment for 24 h [37]. Polystyrene was produced by repeating the preparation process of SST without adding sodium styrene sulfonate and drying it at 80 °C.

### 4.3. Methods

#### 4.3.1. Drilling Fluid and Hot Rolling

The based bentonite fluid served as the fundamental drilling fluid in this study. Before adding it to the drilling fluid, SST was stirred at 4000–6000 rpm for 20 min. For hot rolling, the dispersion was loaded into an aging tank and sealed. The aging tank was then placed into the roller heating furnace and rolled for 16 h under the preset temperature.

#### 4.3.2. Characterization of SST

SST was characterized by Fourier Transform Infrared Spectroscopy (FT-IR, Shimadzu IRTracer-100, Niigata, Japan) with a scanning frequency of 400 cm^−1^–4000 cm^−1^. The SST sample was dried at 80 °C until no weight change occurred and utilized in thermal stability analysis, which was performed using a thermogravimetric analyzer (Mettler Toledo TGA2, Zurich, Switzerland) at 40–800 °C with a heating rate of 10 K/min. The composition of SST was derived from the elemental analyzer (Elementar Unicube, Heraeus, Germany).

SST with a concentration of 0.25 wt% was dropped on the silicon wafer and dried, and then the microscopic morphology of the microspheres SST was observed using scanning electron microscopy at 10,000×. Additionally, the microscopic morphology of the filter cake was observed at 3000×. The sample preparation procedure followed: we dried the filter cake and glued it on the carrier table using conductive adhesive before gold spraying.

#### 4.3.3. Particle Size and Zeta Potential

The particle size of SST and the drilling fluids was determined using dynamic light scattering (DLS) by a particle sizer (Malvern Mastersizer 3000, Marvin, UK) at 10–12% shading. Zeta potential was measured using a potential analyzer (Malvern Zetasizer Nano Z, Marvin, UK) with a 1000 dilution of the sample. The zeta potential value of each sample was achieved by averaging three test results to minimize instrumental measurement errors.

#### 4.3.4. Evaluation of Plugging Performance

The synthetic sandstone core made by the Key Laboratory of Unconventional Oil and Gas Resources Development of China University of Petroleum (East China) was utilized to evaluate the plugging performance of SST. The core was mounted into a core-flooding apparatus (LDY50-180A, Suzhou, China) and entirely saturated with water. Water flooding was conducted with an injection rate of 5 mL/min for 48 h. The confining pressure of the flooding experiments was 5 MPa, and the maximum injection pressure was kept below 5 MPa. After completing water flooding, the core was dried using a vacuum oven at 105 °C for 48 h. Following the above experimental procedure, the core-flooding was performed for the flooding experiment using SST with a 2% (volumetric) concentration. The water saturation of the synthetic core after water and SST flooding was tested by NMR (MacroMR12-150H, Suzhou, China) to determine the initial porosity of the core and evaluate the plugging effect of SST, respectively.

#### 4.3.5. Evaluation of Drilling Fluid Performance

The elastic modulus (G′) and viscous modulus (G″) of the drilling fluid at different shear frequencies, as well as the viscosity at different shear rates, were measured by a Harker rheometer (Physica MCR301, Austria). The shear frequency ranged from 0.013 Hz, while the shear rate ranged from 0.1–1000 1/s.

The fluid loss was measured based on API standards. Specifically, filtration loss or fluid loss is the volume of water lost by drilling fluid through a 75 mm diameter filter area (filter paper) within 30 min at room temperature and 100 psi differential pressure [2,24,28]. The experimental instrument is the medium-pressure filtration meter (SD3, Qingdao Tongchun Oil Instrument Co, Ltd., Qingdao, China). The filter cake was achieved from the mud cake on the filter paper after 30 min of filtration.

The drilling fluid was diluted 10 times and tested for suspension stability using an emulsion stability analyzer (Formulation Turbiscan Lab, Toulouse, France) with a scanning interval of 5 min for 2 h [39,40]. The suspension stability of the drilling fluid was analyzed by examining the variation in transmittance of a 20 mL drilling fluid over time.

## Data Availability

Data available on request due to privacy.
